# Subdivision of M category for nasopharyngeal carcinoma with synchronous metastasis: time to expand the M categorization system

**DOI:** 10.1186/s40880-015-0031-9

**Published:** 2015-08-12

**Authors:** Lu-Jun Shen, Si-Yang Wang, Guo-Feng Xie, Qi Zeng, Chen Chen, An-Nan Dong, Zhi-Mei Huang, Chang-Chuan Pan, Yun-Fei Xia, Pei-Hong Wu

**Affiliations:** Department of Medical Imaging and Interventional Radiology, Sun Yat-sen University Cancer Center, State Key Laboratory of Oncology in South China, Collaborative Innovation Center for Cancer Medicine, Guangzhou, Guangdong 510060 P. R. China; Department of Radiation Oncology, The Fifth Affiliated Hospital of Sun Yat-sen University, Zhuhai, Guangdong 519000 P. R. China; Department of Radiation Oncology, Cancer Center of Guangzhou Medical University, Guangzhou, Guangdong 510080 P. R. China; Department of Radiation Oncology, Sun Yat-sen University Cancer Center, State Key Laboratory of Oncology in South China, Collaborative Innovation Center for Cancer Medicine, Guangzhou, Guangdong 510060 P. R. China; Department of Medical Oncology, Sichuan Cancer Hospital and Institute, Chengdu, Sichuan 610041 P. R. China

**Keywords:** Nasopharyngeal carcinoma, TNM staging, Metastasis, Prognosis

## Abstract

**Introduction:**

The current metastatic category (M) of nasopharyngeal carcinoma (NPC) is a “catch-all” classification, covering a heterogeneous group of tumors ranging from potentially curable to incurable. The aim of this study was to design an M categorization system that could be applied in planning the treatment of NPC with synchronous metastasis.

**Methods:**

A total of 505 NPC patients diagnosed with synchronous metastasis at Sun Yat-sen University Cancer Center between 2000 and 2009 were involved. The associations of clinical variables, metastatic features, and a proposed M categorization system with overall survival (OS) were determined by using Cox regression model.

**Results:**

Multivariate analysis showed that Union for International Cancer Control (UICC) N category (N1–3/N0), number of metastatic lesions (multiple/single), liver involvement (yes/no), radiotherapy to primary tumor (yes/no), and cycles of chemotherapy (>4/≤4) were independent prognostic factors for OS. We defined the following subcategories based on liver involvement and the number of metastatic lesions: M1a, single lesion confined to an isolated organ or location except the liver; M1b, single lesion in the liver and/or multiple lesions in any organs or locations except the liver; and M1c, multiple lesions in the liver. Of the 505 cases, 74 (14.7%) were classified as M1a, 296 (58.6%) as M1b, 134 (26.5%) as M1c, and 1 was not specified. The three M1 subcategories showed significant difference in OS [M1b vs. M1a, hazard ratio (HR) = 1.69, 95% confidence interval (CI) = 1.16–2.48, *P* = 0.007; M1c vs. M1a, HR = 2.64, 95% CI = 1.75–3.98, *P* < 0.001].

**Conclusions:**

We developed an M categorization system based on the independent factors related to the prognosis of patients with metastatic NPC. This system may be helpful to further optimize individualized care for NPC patients.

## Background

The tumor-node-metastasis (TNM) staging system, which describes the anatomic extent of cancer, has been widely used to aid clinicians and investigators in planning treatment, assessing prognosis, and facilitating communication [1, 2]. Recent years, great progress in diagnostic imaging and radiation techniques for nasopharyngeal carcinoma (NPC) has been developed, and a series of modifications have been introduced to the TNM staging system that focuses on the primary tumor (T) and local node (N) descriptors [3–7]. By contrast, the current metastasis (M) category is still a “catch-all” classification, covering a heterogeneous group of NPCs whose outlooks range from potentially curable to incurable [8–11].

There is emerging evidence that the anatomic extent of metastasis closely associates with the prognosis of patients with metastatic NPC. Liver metastasis seems to be an independent negative prognostic factor versus bone or lung metastasis, whereas lung metastasis alone appeared to be a relatively favorable prognostic factor [12–14]. Single metastatic lesion in isolated location (organ or site) was reported to associate with prolonged survival versus multiple metastatic lesions in isolated or multiple locations [14–16]. Moreover, a growing body of evidence showed that long-term survival could be achieved for selective NPC patients with limited metastatic lesions by a combination of systemic and local therapies [17, 18]. These findings suggest that further subdivision of the M category for metastatic NPC may be necessary to aid clinicians in assessing the prognosis and planning the treatment.

Due to the rarity of synchronous metastatic NPC patients, most published studies included both patients with synchronous and those with metachronous metastasis, or focused only on the patients with metachronous metastatic NPC in their analysis. Only one study specifically evaluated the prognostic values of metastatic features in patients with synchronous metastatic NPC and identified no significant findings in multivariate analysis, which may partly result from sample size limitations [14]. Because NPC patients with synchronous metastasis underwent different treatment regimens and had different survival rates versus those with metachronous metastasis [12, 19], a detailed analysis of the data of metastatic NPC based on a large cohort of patients with synchronous metastasis is warranted.

In this study, we set out to obtain a detailed analysis of data related to synchronous metastatic NPC and to design an M categorization system that is simple and useful for the best individualized care for these patients. In addition, the implication of this system in the management of primary NPC was also assessed.

## Patients and methods

### Patient selection

The medical records of 1,647 NPC patients with distant metastasis treated at the Sun Yat-sen University Cancer Center (SYSUCC) between January 2000 and December 2009 were reviewed. The inclusion criteria were as follows: (1) histologically confirmed NPC with distant metastasis at initial diagnosis and (2) presence of pre-treatment evaluation including complete history, physical examination, hematology and biochemistry profiles, computed tomography (CT) or magnetic resonance imaging (MRI) scans of the head and neck regions, radiographs/CT scans of the chest, sonography/CT scans of the abdomen, and whole-body bone scan. The exclusion criteria are any of the following: (1) refusal of treatment and (2) presence of other malignancies. The Hospital Ethics Committee in SYSUCC approved this study.

### Variables and staging workup

A multidisciplinary team consisting of radiation oncologists, radiologists, and oncologists assembled to review the medical charts and imaging data of the metastatic NPC patients, with a special focus on the anatomic extent of metastasis at the initial diagnosis. The metastatic features assessed included the number of metastatic locations (isolated vs. multiple), the involvement of specific metastatic locations, and the number of metastases in each metastatic location (single vs. multiple). A new system of M category was hereafter proposed, with the variables described above taken into account.

The T and N categories of the primary NPC were staged according to the 7th edition of International Union against Cancer (UICC) staging system. Additional variables assessed included patient characteristics [sex, age, Karnofsky performance score (KPS), and body mass index (BMI)] and treatment (cycles of chemotherapy and radiotherapy for primary tumor).

### Treatment and follow-up

All patients received palliative chemotherapy as a systemic treatment after admission. The first-line regimen was nearly exclusively platinum-based, with cisplatin in combination with one or two of the following drugs: 5-fluorouracil, paclitaxel, gemcitabine, and bleomycin for 4–6 cycles. Treatment was discontinued by request of the patients or for intolerable drug toxicity; the median number of cycles was 4 (range 1–27). Local therapies such as surgery, radiotherapy, interventional embolization, and radiofrequency ablation served as options for those who still had metastatic lesions after chemotherapy.

### Follow-up and end point

Patients were followed up and evaluated for their response to therapy every two cycles during systemic chemotherapy and then every 3 months until death. The median follow-up period was 20 months (range 1–120 months). Survival status was verified on August 31, 2014 by direct telecommunication with the patient or their family and by checking the clinic attendance records. The primary outcome was overall survival (OS), which was defined as the time from diagnosis of distant metastasis to death by any causes or the last follow-up.

### Statistical analysis

Wilcoxon rank sum and Chi square tests were used to compare ordinal and categorical variables between three M1 subcategories, respectively. The Kaplan–Meier method was used to estimate the OS, and the estimated survival curves for different groups were compared by using the log-rank test. All of the covariates that were significantly associated with OS were introduced into the backward Cox regression model to determine the independent prognostic factors. Stratified analysis by the proposed M1 subcategories in a multiple-adjusted Cox model was further conducted to investigate the association between primary radiotherapy and prognosis, with covariates including age, UICC N category, and cycles of chemotherapy. A two-tailed *P* value <0.05 was considered significant. Statistical analysis was performed using SPSS 20.0 software (IBM SPSS Inc., Chicago, IL, USA).

## Results

### Patient characteristics

A total of 505 NPC patients were involved in this study. The baseline characteristics of patients are shown in Table 1. The median age was 48 years (range 18–78 years). Among the patients, 427 (84.6%) were males, and 78 (15.4%) were females; 468 (92.7%) had undifferentiated non-keratinizing carcinoma, 29 (5.7%) had differentiated non-keratinizing carcinoma, and 8 (1.6%) had keratinizing squamous cell carcinoma; 306 (60.6%) had isolated metastasis, and 199 (39.4%) had widespread metastasis. The most frequently involved locations for metastases were the bones (65.9%), the liver (30.7%), distant lymph nodes (28.5%), and the lungs (26.9%); isolated organ metastasis was common among the bone (65.9%), the lung (41.2%), and the liver (36.1%); and the metastasis was rare in distant lymph nodes (6.9%). Multiple lesions were detected more frequently than single lesions for all the involved organs or locations (Table 2).

**Table 1:** Baseline characteristics of 505 nasopharyngeal carcinoma (NPC) patients with synchronous metastasis

Characteristic	Entire group	M1 subcategory^a^			*P* value
		M1a	M1b	M1c	
Total	505	74	296	134	
Age (years)					0.426
<48	259 (51.3)	43 (58.1)	149 (50.3)	66 (49.3)	
≥48	246 (48.7)	31 (41.9)	147 (49.7)	68 (50.7)	
Sex					0.212
Male	427 (84.6)	58 (78.4)	256 (86.5)	112 (83.6)	
Female	78 (15.4)	16 (21.6)	40 (13.5)	22 (16.4)	
UICC T category					0.756
T1	25 (5.0)	3 (4.1)	13 (4.4)	8 (6.0)	
T2	88 (17.4)	14 (18.9)	47 (15.9)	27 (20.1)	
T3	227 (45.0)	29 (39.2)	139 (47.0)	59 (44.0)	
T4	165 (32.7)	28 (37.8)	97 (32.8)	40 (29.9)	
UICC N category					0.143
N0	28 (5.5)	7 (9.5)	18 (6.1)	3 (2.2)	
N1	160 (31.7)	25 (33.8)	90 (30.4)	44 (32.8)	
N2	227 (45.0)	35 (47.3)	128 (43.2)	64 (47.8)	
N3	90 (17.8)	7 (9.5)	60 (20.3)	23 (17.2)	
KPS					0.201^b^
≥80	476 (94.3)	73 (98.6)	276 (93.2)	126 (94.0)	
<80	29 (5.7)	1 (1.4)	20 (6.8)	8 (6.0)	
BMI					0.699
≥18.5	430 (85.1)	65 (87.8)	249 (84.1)	115 (85.8)	
<18.5	75 (14.9)	9 (12.2)	47 (15.9)	19 (14.2)	
Radiotherapy to primary tumor					<0.001
No	267 (52.9)	22 (29.7)	147 (49.7)	98 (73.1)	
Yes	238 (47.1)	52 (70.3)	149 (50.3)	36 (26.9)	
Cycles of chemotherapy					0.864
≤ 4	273 (54.1)	42 (56.8)	159 (53.7)	71 (53.0)	
>4	232 (45.9)	32 (43.2)	137 (46.3)	63 (47.0)	

All values are presented as numbers of patients followed by percentages in the parentheses.

*UICC* International Union Against Cancer, *KPS* Karnofsky performance score and *BMI* body mass index.

^a^One of the 505 patients with an unspecified metastatic disease cannot be classified to any of the three M1 subcategories.

^b^Fisher’s exact test was used; *P* < 0.05 was considered significant.

**Table 2:** Location of metastases and characteristics within the entire cohort of NPC patients

Location and type of metastases	Number of patients (cases [%])^a^	Percentage (%)^b^
Bone		
Isolated bone metastasis	184 (65.9)	
Total bone metastases	333 (100.0)	65.9
Number of lesions		
Single metastasis	74 (22.2)	
Multiple metastases	239 (71.8)	
Not specified	20 (6.0)	
Lung		
Isolated lung metastasis	56 (41.2)	
Total lung metastases	136 (100.0)	26.9
Number of lesions		
Single metastasis	42 (30.9)	
Multiple metastases	89 (65.4)	
Not specified	5 (3.7)	
Liver		
Isolated liver metastasis	56 (36.1)	
Total liver metastases	155 (100.0)	30.7
Number of lesions		
Single metastasis	43 (27.7)	
Multiple metastases	102 (65.8)	
Not specified	10 (6.5)	
Distant lymph nodes		
Isolated lymph node metastases	10 (6.9)	
Total lymph node metastases	144 (100.0)	28.5
Number of lesions		
Single metastasis	15 (10.4)	
Multiple metastases	112 (77.8)	
Not specified	17 (11.8)	
Others^c^		
Isolated metastasis	0 (0.0)	
Total number of other metastases	12 (100.0)	2.4
Number of lesions		
Single metastasis	4 (33.3)	
Multiple metastases	2 (16.7)	
Not specified	6 (50)	

^a^The percentages of patients with respect to the total of patients with metastasis at the corresponding locations.

^b^The percentage of patients with respect to the total of patients with synchronous metastatic NPC.

^c^Others include the spleen, kidney, pleura, breast gland, abdominal wall, and thyroid gland.

### M category subdivision and survival

**Figure 1:**
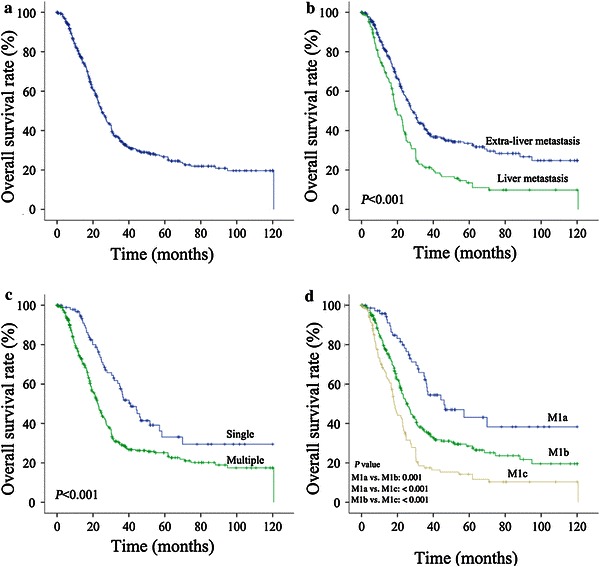
Kaplan–Meier curves of overall survival (OS) in patients with nasopharyngeal carcinoma (NPC). **a** the entire cohort of NPC patient; **b** the liver metastasis and extra-liver metastasis groups; **c**,the groups with single or multiple metastatic lesions; **d** the groups in different M1 subcategories as proposed.

Overall, 312 patients (61.8%) died before the last follow-up. The median OS time was 24.9 months (range 1–120 months), and the 1-, 3-, and 5-year OS rates were 80.0%, 34.9%, and 27.0%, respectively, for the whole population (Figure 1a).

The metastatic locations, number of metastatic locations, and number of metastatic lesions were analyzed separately to identify the optimal grouping strategy (Table 3). Univariate analysis showed that the involvement of the liver (*P* < 0.001) and multiple lesions (*P* < 0.001) were significantly associated with an unfavorable OS among patients with synchronous metastasis (Figure 1b, c). Other factors that significantly associated with OS included age, UICC N category, KPS, radiotherapy to primary tumor, and cycles of chemotherapy.

**Table 3:** Overall survival (OS) according to the locations, number of locations, and number of lesions in each location

Category and variable	Number of patients^a^	Median OS (months)	3-year OS rate (%)	*P* value
Isolated metastasis versus multiple metastases				
Number of metastatic locations				0.001
Multiple locations	199	22.4	24.0	
Isolated location	306	27.4	41.6	
Number of metastatic lesions in each location				<0.001
Multiple lesions	410	22.8	29.5	
Isolated lesion	95	41.0	57.6	
Involved locations				
Bone involvement				0.404
Yes	333	26.2	35.8	
No	172	23.9	33.5	
Lung involvement				0.559
Yes	136	25.1	30.3	
No	369	24.6	36.6	
Liver involvement				<0.001
Yes	155	19.7	21.7	
No	350	28.4	41.1	
Distant LN involvement				0.104
Yes	144	23.2	27.6	
No	361	26.7	37.6	
Number of lesions and locations				
Isolated bone metastasis				0.065
Single lesion	50	44.2	60.7	
Multiple lesions	133	25.0	38.6	
Isolated lung metastasis				0.115
Single lesion	20	57.0	62.1	
Multiple lesions	36	27.2	30.9	
Isolated liver metastasis				0.473
Single lesion	21	25.6	43.6	
Multiple lesions	35	17.8	22.4	
Isolated distant LN metastasis				–
Single lesion	4	63.7	75.0	
Multiple lesions	6	21.0	54.5	
Total isolated metastasis^b^				0.002
Single lesion	95	41.0	57.6	
Multiple lesions	210	23.3	34.6	

*LN* lymph node.

^a^The total number of patients does not coincide exactly with the corresponding number in Table 2 because the number of lesions for some patients is not specified.

^b^These include isolated metastasis in the bones, the lungs, the liver, and distant lymph node.

Multivariate analysis using a backward method and including all of the significant prognostic factors mentioned above indicated that UICC N category (N1–3/N0, *P* = 0.031), number of metastatic lesions (multiple/single, *P* = 0.005), liver involvement (yes/no, *P* < 0.001), radiotherapy to primary tumor (yes/no, *P* < 0.001), and cycles of chemotherapy (>4/≤4, *P* < 0.001) were independent prognostic factors for patients with synchronous metastatic NPC (Table 4). Based on the two independent prognostic factors, liver involvement and number of metastatic lesions, we further subdivided the entire cohort of NPC into three M1 subcategories: M1a, single lesion confined to an isolated organ or location (the liver excluded); M1b, single lesion in the liver and/or multiple lesions in any organs or locations except for the liver; and M1c, multiple lesions in the liver. Of all the patients, 74 (14.7%) were in the M1a subcategory, 296 (58.6%) were in M1b, and 134 (26.5%) were in M1c, with 1 patient not specified. The median OS time for M1a, M1b, and M1c subcategories were 46.0, 25.1, and 18.3 months, respectively, and the 3-year OS rates were 62.1%, 36.1%, 17.9%, respectively (Figure 1d). Multivariate analysis suggested that different M1 subcategories showed significant difference regarding OS [M1b vs. M1a: hazard ratio (HR) = 1.69, 95% confidence interval (CI) = 1.16–2.48, *P* = 0.007; M1c vs. M1a: HR = 2.64, 95% CI = 1.75–3.98, *P* < 0.001] (Table 5).

**Table 4:** Independent prognostic factors from multivariate analysis for OS

Variable	HR	95% CI	*P* value
Age (≥48 years/<48 years)	1.24	0.99–1.57	0.059
UICC N stage (N1–3/N0)	1.84	1.06–3.19	0.031
Number of metastatic lesions (multiple/single)	1.60	117–2.20	0.005
Liver involvement (yes/no)	1.56	1.23–1.96	<0.001
Cycles of chemotherapy (>4/≤4)	0.63	0.50–0.79	<0.001
Radiotherapy to primary tumor (yes/no)	0.59	0.47–0.75	<0.001

*UICC* International Union Against Cancer, *HR* hazard ratio, *CI* confident interval.

**Table 5:** Univariate and multivariate analysis in assessing the impact of M subcategories for patients with synchronous metastatic NPC

Variable	HR	95% CI	*P*
Univariate analysis			
Age (≥48 years/<48 years)	1.32	1.05–1.65	0.016
Sex (female/male)	0.85	0.62–1.17	0.319
UICC T category (T3–4/T1–2)	0.93	0.79–1.22	0.930
UICC N category (N1–3/N0)	1.93	1.12–3.31	0.017
KPS (< 80/≥ 80)	1.66	1.03–2.67	0.036
BMI (<18.5/≥18.5)	1.23	0.90–1.67	0.189
Radiotherapy to primary tumor (yes/no)	0.52	0.41–0.65	<0.001
Cycles of chemotherapy (>4/≤4)	0.71	0.57–0.89	0.003
M subcategory^a^			<0.001
M1a	Ref		
M1b	1.90	1.30–2.76	0.001
M1c	3.10	2.08–4.62	<0.001
Multivariate analysis			
Age (≥48 years/<4 years)	1.24	0.99–1.56	0.065
UICC N category (N1–3/N0)	1.83	1.05–3.17	0.032
Radiotherapy to primary tumor (yes/no)	0.63	0.49–0.80	<0.001
Cycles of chemotherapy (>4/≤4)	0.63	0.50–0.79	<0.001
M subcategory^a^			<0.001
M1a	Ref		
M1b	1.69	1.16–2.48	0.007
M1c	2.64	1.75–3.98	<0.001

*KPS* Karnofsky performance score; *BMI* body mass index; *Ref* reference; Other abbreviations as in Table 4.

^a^One of the 505 patients with an unspecified metastatic disease cannot be classified to any of the three subcategories.

### Radiotherapy for primary NPC and M1 subcategories

Of the 505 patients, 238 (47.1%) received radiotherapy for primary NPC, with a total external radiation doses ranged from 60 to 78 Gy (median, 72 Gy). A significant difference in the distribution of radiotherapy to primary tumors was observed between three M1 subcategories. Therefore, a further stratified analysis was conducted to determine the impact of primary radiotherapy on OS. Multiple-adjusted model including age, UICC N category, cycles of chemotherapy, and radiotherapy to primary tumor indicated that radiotherapy to the primary tumor was associated with an improved OS among patients with M1b (HR = 0.69, 95% CI = 0.51–0.94, *P* = 0.017) and M1c (HR = 0.43, 95% CI = 0.25–0.74, *P* = 0.002) tumors. In the M1a subcategory, such association was not found significant (HR = 0.86, 95% CI = 0.38–1.94, *P* = 0.716).

## Discussion

Our study provided several notable findings: (1) among 505 NPC patients with synchronous metastasis, the most frequently involved organs or sites at diagnosis were the bones (65.9%), the liver (30.7%), distant lymph nodes (28.5%), and the lungs (26.9%), respectively; (2) UICC N category, number of metastatic lesions, liver involvement, cycles of chemotherapy, and radiotherapy to primary tumors were independently associated with the OS of patients with synchronous metastatic NPC; and (3) based on liver involvement and number of metastatic lesions, we proposed a new M categorization system to further subdivide the population into three M1 subcategories, which showed a high degree of difference regarding OS and have important implications in the management of the metastatic disease.

There has been only one report that has specifically evaluated the data related to synchronous metastatic NPC. Pan *et al*. [14] retrospectively analyzed the data of 376 NPC patients with synchronous metastasis, and the results in univariate analysis suggested that both liver involvement and the presence of multiple lesions were unfavorable factors for OS. However, these two factors failed to reach significance in multivariate analysis, which may possibly be explained by the relatively small sample size and the heterogeneity of the involved population, with the admission time ranging from 1995 to 2007 [14]. Therefore, in this study, we conducted a detailed analysis based on a large cohort of NPC patients with synchronous metastases admitted to our center between 2000 and 2009.

The current study introduced the clinical course of synchronous metastasis in a large cohort of NPC patients treated in the contemporary era. The OS time after metastasis ranged from 1 to 120 months, indicating that long-term survival is possible in certain proportions of patients with metastases. The median OS time in our study was 24.9 months, which was close to the estimated 25 months reported by Lin *et al*. [20] and 22 months reported by Li *et al*. [16].

Liver involvement was reported to associate with an unfavorable prognosis [21, 22], and lung metastasis alone was a favorable prognostic factor among patients with metachronous metastatic NPC [13]. By contrast, few studies addressed the issue of the prognostic values of metastatic locations among patients with synchronous metastatic NPC. In our study, a significant difference in OS time was found between patients with metastatic NPC with and without liver involvement (21.7 vs. 41.1 months, *P* < 0.001), whereas no significant difference was found between patients with lung metastasis alone and those with bone metastasis alone or distant lymph nodal metastasis alone. As liver metastasis has been conventionally regarded as an indicator of poor prognosis among NPC patients, the treatment has largely been palliative [8, 23]. In recent years, several studies showed that CT-guided radiofrequency ablation (RFA) can be performed with a high degree of technical effectiveness and offer the promise of prolonged survival time in selected NPC patients with liver metastases [17, 18]. However, these results must be interpreted with caution and future prospective studies with a large cohort are needed to validate these findings.

The association between the number of metastatic lesions (single/multiple) and OS has been extensively studied and demonstrated to be significant in NPC patients with synchronous and metachronous metastases by a series of studies [14, 15], whereas its prognostic value as compared with the number of metastatic locations remains unknown. It is intriguing that in our study, both the number of metastatic lesions (single/multiple) and the number of metastatic locations (isolated/multiple) were significant associated with OS in univariate analysis. A multivariate analysis that included all of the significant covariates suggested that the number of metastatic lesions, but not the number of metastatic locations, was an independent prognostic factor for OS. These results indicated that patients with single metastatic lesion need special attention. As there is enormous evidence that NPC patients with single metastatic lesion in isolated organ, such as the lung [17, 24], the liver [25], and the bone [26], can benefit from combined local and systemic therapies [27], an accurate imaging diagnosis for NPC patients with limited metastatic lesions will be of vital importance in identifying this group of patients and providing individualized treatment.

A major challenge we face with the TNM staging system is how to modify the M categorization system for a more precise prognostic prediction and treatment planning. Many studies have reported that the greater the tumor load, the worse the prognosis in NPC [28, 29]. We have proposed a theoretical formula for the assessment of metastatic NPC, *V*_t_ = *V*_1_ + *V*_2_ + *V*_3_ +, …, + *V*_n_ + *V*_x_, where *V*_1_, *V*_2_, *V*_3_, …, and *V*_n_ are defined as the tumor volume of each visible lesion under the current best diagnostic imaging system, and *V*_x_ is defined as the total tumor volume of invisible lesions [30]. The ideal strategy in the management of metastatic NPC is to eliminate all the visible lesions to achieve complete remission (CR) by combined local and systemic therapies and then to eradicate invisible lesions (*V*_x_) by using chemotherapy, immunotherapy, or targeted therapy. However, this sophisticated system that localizes and targets every visible metastatic lesion is difficult to practice due to the restrictions in current diagnostic and treatment techniques. Hence, an M categorization system with a delicate balance of accuracy and practicality should be considered. Practicality requires that a new category strategy shall be relevant to current clinical practice, be evidence-based, and reflect the dominant prognostic factors consistently identified in Cox multivariate regression analyses. Based on our results and a review of the published literature, we propose to subdivide the status of synchronous metastasis of NPC into three M1 subcategories. The advantage of this proposed M categorization system is that it can differentiate patients with drastically different prognoses and emphasize a more active way to manage patients with single metastatic lesion. This system may also be useful in the design of clinical trials and help standardize the reported results of any therapeutic interventions.

A major controversy exists concerning the necessity of treating the primary NPC with an optimal treatment, especially for patients with distant metastases involving the liver or multiple organs or sites [20, 31]. Since 2011, concurrent chemoradiotherapy was suggested as a choice for selected patients (patients with distant metastases in limited sites or with a small tumor burden, or patients with symptoms in the primary or any nodal site) in the National Comprehensive Cancer Network (NCCN) guidelines. In more recent years, Tian *et al*. [15] retrospectively analyzed the prognosis of 85 NPC patients initially presenting with liver metastasis and found that radiotherapy for the primary tumor could significantly prolong survival time (no/yes: HR = 2.87, 95% CI = 1.61–5.10, *P* < 0.001), and 5 patients achieved long-term disease-free survival after undergoing radiotherapy for the primary lesion. Consistent with their study, our study showed that primary radiotherapy was independently associated with prolonged OS for patients with M1b and M1c NPC. These findings revealed that a considerable proportion of patients with extensive distant metastases could benefit from radiotherapy for primary NPC, and further studies are needed to identify the targeted patients.

Our study has several limitations. First, it is a retrospective study and the cohort was obtained from a specific, regionally based population that may be not representative of the general population of NPC patients with synchronous metastases. Second, the modes of chemotherapy and radiotherapy applied varied, which might have a confounding effect. Third, the serum Epstein-Barr virus (EBV) DNA level has been demonstrated to be an important prognostic factor among patients with recurrent/metastatic NPC [32]; however, we failed to include it in our analysis due to the lack of data at diagnosis in our cancer center (116 patients, 23.0%). The prognostic value of serum EBV DNA level and its association with the anatomical extent of the metastasis of NPC should be further assessed. Finally, the metastases of NPC in most patients involved in our study were clinically diagnosed, and only a small proportion (44 patients, 8.7%) had pathologic confirmation, which could be a potential source of bias. For these reasons, we must validate our findings in a multi-institutional prospective study in the future.

## Conclusions

We developed an M categorization system based on the independent prognostic factors related to the metastasis of NPC in patients. Multi-institutional external validation of this categorization system is warranted in the future.

## References

[1] Greene FL, Sobin LH (2008). The staging of cancer: a retrospective and prospective appraisal. CA Cancer J Clin.

[2] Gospodarowicz MK, Miller D, Groome PA, Greene FL, Logan PA, Sobin LH (2004). The process for continuous improvement of the TNM classification. Cancer.

[3] Ng WT, Yuen KT, Au KH, Chan OS, Lee AW (2014). Staging of nasopharyngeal carcinoma—the past, the present and the future. Oral Oncol.

[4] OuYang PY, Su Z, Ma XH, Mao YP, Liu MZ, Xie FY (2013). Comparison of TNM staging systems for nasopharyngeal carcinoma, and proposal of a new staging system. Br J Cancer.

[5] Shi Q, Shen C, Kong L, Wang X, Ding J, Gao Y (2014). Involvement of both cervical lymph nodes and retropharyngeal lymph nodes has prognostic value for N1 patients with nasopharyngeal carcinoma. Radiat Oncol.

[6] Yang Z, Xu JM, Gong JS, Ma J, Yu JB, Zhou SP (2013). Relationship between dynamic contrast-enhanced and perfusion magnetic resonance imaging and T-staging of nasopharyngeal carcinoma. Zhonghua Yi Xue Za Zhi.

[7] Young LS, Dawson CW (2014). Epstein-Barr virus and nasopharyngeal carcinoma. Chin J Cancer.

[8] Fandi A, Bachouchi M, Azli N, Taamma A, Boussen H, Wibault P (2000). Long-term disease-free survivors in metastatic undifferentiated carcinoma of nasopharyngeal type. J Clin Oncol.

[9] Chou CW, Liu JM, Wu MF, Li AF, Tie CM, Chi KH (1997). Prolonged survival in a nasopharyngeal carcinoma patient with multiple metastases: a case report and review of the literature. Jpn J Clin Oncol.

[10] Wang CT, Cao KJ, Li Y, Xie GF, Huang PY (2007). Prognosis analysis of nasopharyngeal carcinoma patients with distant metastasis. Ai Zheng..

[11] Setton J, Wolden S, Caria N, Lee N (2012). Definitive treatment of metastatic nasopharyngeal carcinoma: report of 5 cases with review of literature. Head Neck.

[12] Ong YK, Heng DM, Chung B, Leong SS, Wee J, Fong KW (2003). Design of a prognostic index score for metastatic nasopharyngeal carcinoma. Eur J Cancer.

[13] Hui EP, Leung SF, Au JS, Zee B, Tung S, Chua D (2004). Lung metastasis alone in nasopharyngeal carcinoma: a relatively favorable prognostic group. A study by the Hong Kong Nasopharyngeal Carcinoma Study Group. Cancer.

[14] Pan CC, Lu J, Yu JR, Chen P, Li W, Huang ZL (2012). Challenges in the modification of the M1 stage of the TNM staging system for nasopharyngeal carcinoma: a study of 1027 cases and review of the literature. Exp Ther Med..

[15] Tian YM, Zeng L, Wang FH, Liu S, Guan Y, Lu TX (2013). Prognostic factors in nasopharyngeal carcinoma with synchronous liver metastasis: a retrospective study for the management of treatment. Radiat Oncol.

[16] Li JX, Huang SM, Wen BX, Lu TX (2014). Prognostic factors on overall survival of newly diagnosed metastatic nasopharyngeal carcinoma. Asian Pac J Cancer Prev.

[17] Pan CC, Wu PH, Yu JR, Li W, Huang ZL, Wang JP (2012). Comparative survival analysis in patients with pulmonary metastases from nasopharyngeal carcinoma treated with radiofrequency ablation. Eur J Radiol.

[18] Jin Y, Cai YC, Cao Y, Cai XY, Tan YT, Shi YX (2012). Radiofrequency ablation combined with systemic chemotherapy in nasopharyngeal carcinoma liver metastases improves response to treatment and survival outcomes. J Surg Oncol.

[19] Teo PM, Kwan WH, Lee WY, Leung SF, Johnson PJ (1996). Prognosticators determining survival subsequent to distant metastasis from nasopharyngeal carcinoma. Cancer.

[20] Lin S, Tham IW, Pan J, Han L, Chen Q, Lu JJ (2012). Combined high-dose radiation therapy and systemic chemotherapy improves survival in patients with newly diagnosed metastatic nasopharyngeal cancer. Am J Clin Oncol.

[21] Katsenos S, Nikolopoulou M (2013). Intramedullary thoracic spinal metastasis from small-cell lung cancer. Monaldi Arch Chest Dis.

[22] Knudson G, Grinis G, Lopez-Majano V, Sansi P, Targonski P, Rubenstein M (1991). Bone scan as a stratification variable in advanced prostate cancer. Cancer.

[23] Pan C, He N, Zhao M, Gu Y, Huang Z, Li W (2011). Subdividing the M1 stage of liver metastasis for nasopharyngeal carcinoma to better predict metastatic survival. Med Oncol.

[24] Ma J, Wen ZS, Lin P, Wang X, Xie FY (2010). The results and prognosis of different treatment modalities for solitary metastatic lung tumor from nasopharyngeal carcinoma: a retrospective study of 105 cases. Chin J Cancer.

[25] Pan C, Wu P, Yu J, Li W, Huang Z, He N (2011). CT-guided radiofrequency ablation prolonged metastatic survival in patients with liver metastases from nasopharyngeal carcinoma. Int J Hyperthermia.

[26] Lim A, Corry J, Lau E, Rischin D (2011). Prolonged remission in a patient with nasopharyngeal carcinoma with a solitary bone metastasis. J Clin Oncol.

[27] Timmerman RD, Bizekis CS, Pass HI, Fong Y, Dupuy DE, Dawson LA (2009). Local surgical, ablative, and radiation treatment of metastases. CA Cancer J Clin.

[28] Chen MK, Chen TH, Liu JP, Chang CC, Chie WC (2004). Better prediction of prognosis for patients with nasopharyngeal carcinoma using primary tumor volume. Cancer.

[29] Chen C, Fei Z, Pan J, Bai P, Chen L (2011). Significance of primary tumor volume and T-stage on prognosis in nasopharyngeal carcinoma treated with intensity-modulated radiation therapy. Jpn J Clin Oncol.

[30] Wu P, Pan C, Gu Y, Huang Z, Zhao M, Gao F (2010). Suggestion of subdividing the M from the TNM classification and personalizing cancer therapy. Zhongguo Zhong Liu.

[31] Yeh SA, Tang Y, Lui CC, Huang EY (2006). Treatment outcomes of patients with AJCC stage IVC nasopharyngeal carcinoma: benefits of primary radiotherapy. Jpn J Clin Oncol.

[32] Chan KC (2014). Plasma Epstein-Barr virus DNA as a biomarker for nasopharyngeal carcinoma. Chin J Cancer.

